# Geophysical monitoring of simulated homicide burials for forensic investigations

**DOI:** 10.1038/s41598-020-64262-3

**Published:** 2020-05-05

**Authors:** Jamie K. Pringle, Ian G. Stimpson, Kristopher D. Wisniewski, Vivienne Heaton, Ben Davenward, Natalie Mirosch, Francesca Spencer, Jon R. Jervis

**Affiliations:** 1 School of Geography, Geology & Environment, Keele University, Keele, Staffordshire ST5 5BG United Kingdom; 2 Department of Criminal Justice and Forensics, School of Law, Policing & Forensics, Science Centre, Staffordshire University, Leek Road, Stoke-on-Trent, ST4 2DF United Kingdom; 3 School of Chemical & Physical Sciences, Keele University, Keele, Staffordshire ST5 5BG United Kingdom; 4 Department of Biochemistry, Carver College of Medicine, University of Iowa, Iowa City, IA 52242 United States of America

**Keywords:** Applied physics, Biophysical chemistry, Hydrology, Environmental monitoring

## Abstract

Finding hidden bodies, believed to have been murdered and buried, is problematic, expensive in terms of human resource and currently has low success rates for law enforcement agencies. Here we present, for the first time, ten years of multidisciplinary geophysical monitoring of simulated clandestine graves using animal analogues. Results will provide forensic search teams with crucial information on optimal detection techniques, equipment configuration and datasets for comparison to active and unsolved cold case searches. Electrical Resistivity (ER) surveys showed a naked burial produced large, low-resistivity anomalies for up to four years, but then the body became difficult to image. A wrapped burial had consistent small, high-resistivity anomalies for four years, then large high-resistivity anomalies until the survey period end. Ground Penetrating Radar (GPR) 110–900 MHz surveys showed the wrapped burial could be detected throughout. 225 MHz GPR data was optimal, but the naked burial was poorly imaged after six years. Results suggested conducting both ER and GPR surveys if the burial style was unknown when searching for interred remains. Surveys in winter and spring produced the best datasets, and, as post-burial time increases, surveying in these seasons became increasingly important. This multidisciplinary study provides critical new insights for law enforcement and families of the disappeared worldwide.

## Introduction

Available statistics for missing persons globally vary. For example, in the United Kingdom, ~250,000 are reported missing every year, but, of those, only ~2,500 are still missing after a year^1^. In the United States, ~650,000 are reported missing every year, but only ~90,000 are still missing after a year^2^. Whilst these still missing numbers are comparatively small, for the families of the missing it is obviously of crucial importance for them to be found, not only for closure if they have been the victim of a homicide, but also to know that justice for the perpetrator(s) has been served. However, current success rates to find the missing are low, with high profile examples being Madeleine McCann in Portugal and Ben Needham in Greece, sadly both presumed dead.

Forensic search methods vary widely. A search strategist may be involved in a case at an early stage to decide which methods would have the highest probability of search success^3^, but this is not for every case globally, and investigations may not be standardised or indeed different techniques undertaken, depending on local experience^4^. Metal detector search teams^5–7^ and specially-trained victim recovery dogs^7–9^ are both commonly used during initial investigations or as part of phased sequential search programmes.

**Figure 1:**
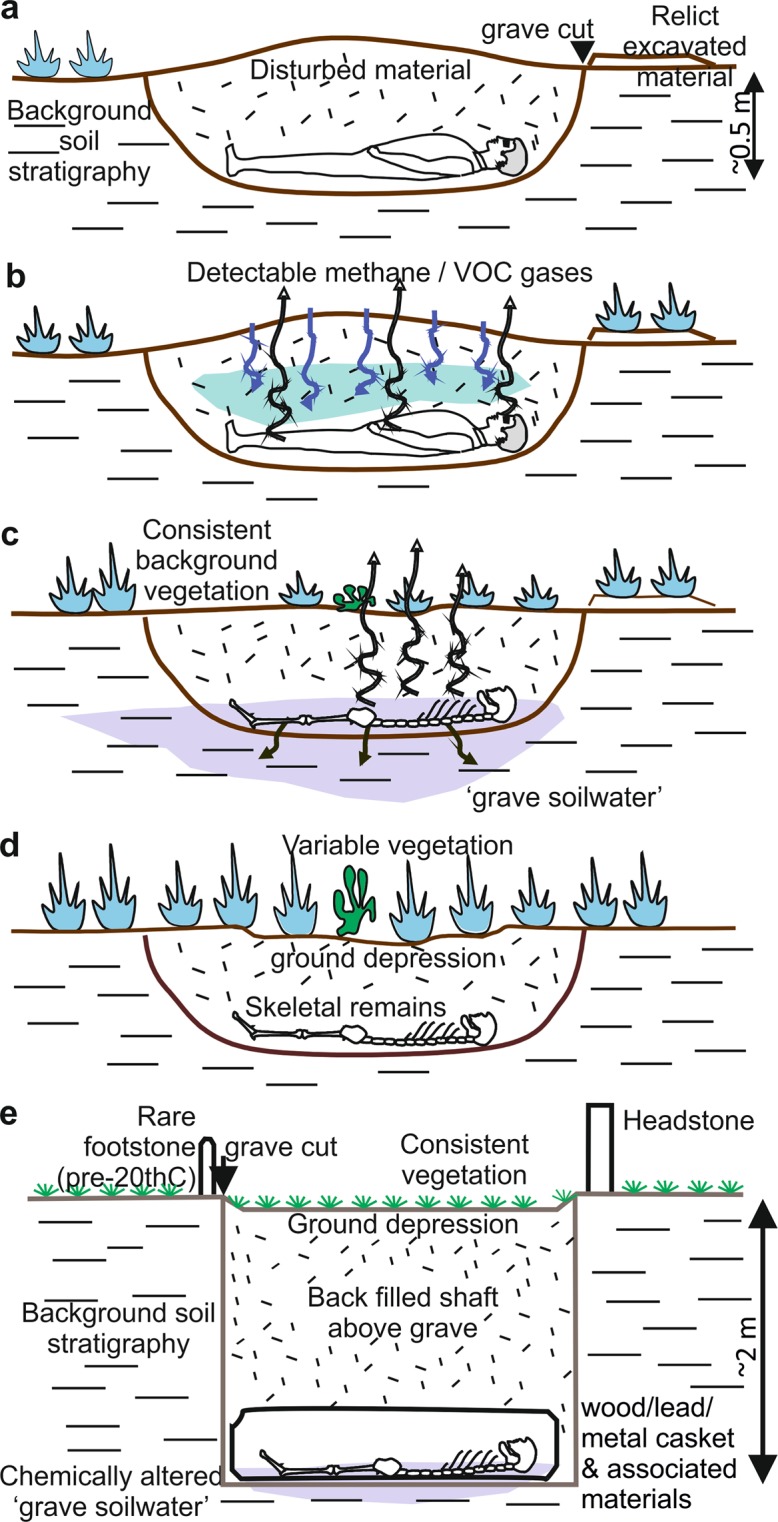
Schematic figures of typical clandestine grave of a homicide victim showing (**a**) just deposited, (**b**) early, (**c**) late and (**d**) skeleton-stage decomposition with respective grave indicators/targets. These contrast with (**e**) isolated graveyard/cemetery earth-cut burials which have quite different style/depths etc. Modified from^14,47^.

Forensic investigators have been increasingly using geoforensic methods in civil or criminal forensic investigations, predominantly to assist search teams as they attempt to locate missing persons or for trace evidence purposes^10–13^. Locating homicide victims buried within clandestine graves is one of the most important and difficult challenges for forensic search teams^3,7,13^. A clandestine grave is defined as an unrecorded burial, often in a remote location, that has been hand-excavated and normally dug <1 m depth below ground level^14^. Due to the circumstances surrounding the event, they are usually rushed in nature, with irregular burial shapes and uneven depths. These graves are quite different than graveyard/cemetery burial styles (Fig. 1), which others have used to determine responses from older graves^14^. Almost half of 87 homicide victims recovered from clandestine burials in the United States were either clothed or encased in material^15^, so these two scenarios were used. One burial contained a naked pig cadaver whilst an adjacent burial contained a pig cadaver wrapped in tarpaulin, the latter frequently used in order to assist with body transportation and concealment. It is, however, emphasised that these do not represent all types of burial style with others^16^ detailing other typical burial scenarios.

Searches typically start from large-scale remote sensing imagery^17,18^, aerial and ultraviolet photography^12,18^, thermal imaging^19^, followed by ground-based vegetation observations^6^, surface geomorphology variation^20^, cadaver search dogs^12^, soil type^3^ and depositional environments^12^, near-surface geophysics^12^, diggability surveys^3^ and ground-probing of anomalous areas^21,22^, before topsoil removal^6^, and finally controlled excavation and potential recovery^7,23^. A typical search will normally only use a subset of these techniques, depending on the individual case being investigated and the associated depositional environment.

Near-surface geophysical methods need a detectable physical contrast between the target and background material^24^ and have been used to locate clandestine graves of homicide victims in criminal search investigations^5,7,25–35^. Geophysical surveys over simulated burials are undertaken to collect control data^36–40^ and to predict what geophysical responses could be in search cases, although the actual response will vary both temporally and between study sites. A few geophysical control surveys have also collected repeat (time-lapse) data^16,29,41–46^. However, at present, a detailed understanding of the temporal persistence of the post burial geophysical anomaly remains unknown due to the time and effort required to collect control data over many years. Geophysical responses from recent (<10 years) clandestine burials are known to vary more than archaeological graves^47^ so an understanding of temporal change is important. Potential reasons for this change could be the modification of grave soil after burial, influence of decomposition products^48^, climatic/weather induced variations of soil moisture content^49^, or indeed burial style – see Fig. 1.

In this study we systematically assess the changing geophysical response of simulated clandestine graves of homicide victims for ten years after burial providing a unique insight into both process and optimum detection technique(s). Unsolved cold cases are typically reviewed every ten years in the United Kingdom so it is crucial to cover this time period. Electrical conductivity of grave and background soil water was monitored *in situ* monthly over six years to quantify site soil water changes. Ground Penetrating Radar (GPR) (110 MHz, 225 MHz, 450 MHz and 900 MHz frequency) 2D profiles and electrical resistivity (surface mapping and 2D imaging) repeat surveys were undertaken quarterly up to six years and then annually up to ten years over the simulated burials (see Supplementary Table S1), in order to determine both their effectiveness for clandestine grave detection over this period, and what was the optimal time to undertake such a forensic geophysical survey.

## Methodology

### Study site

**Figure 2:**
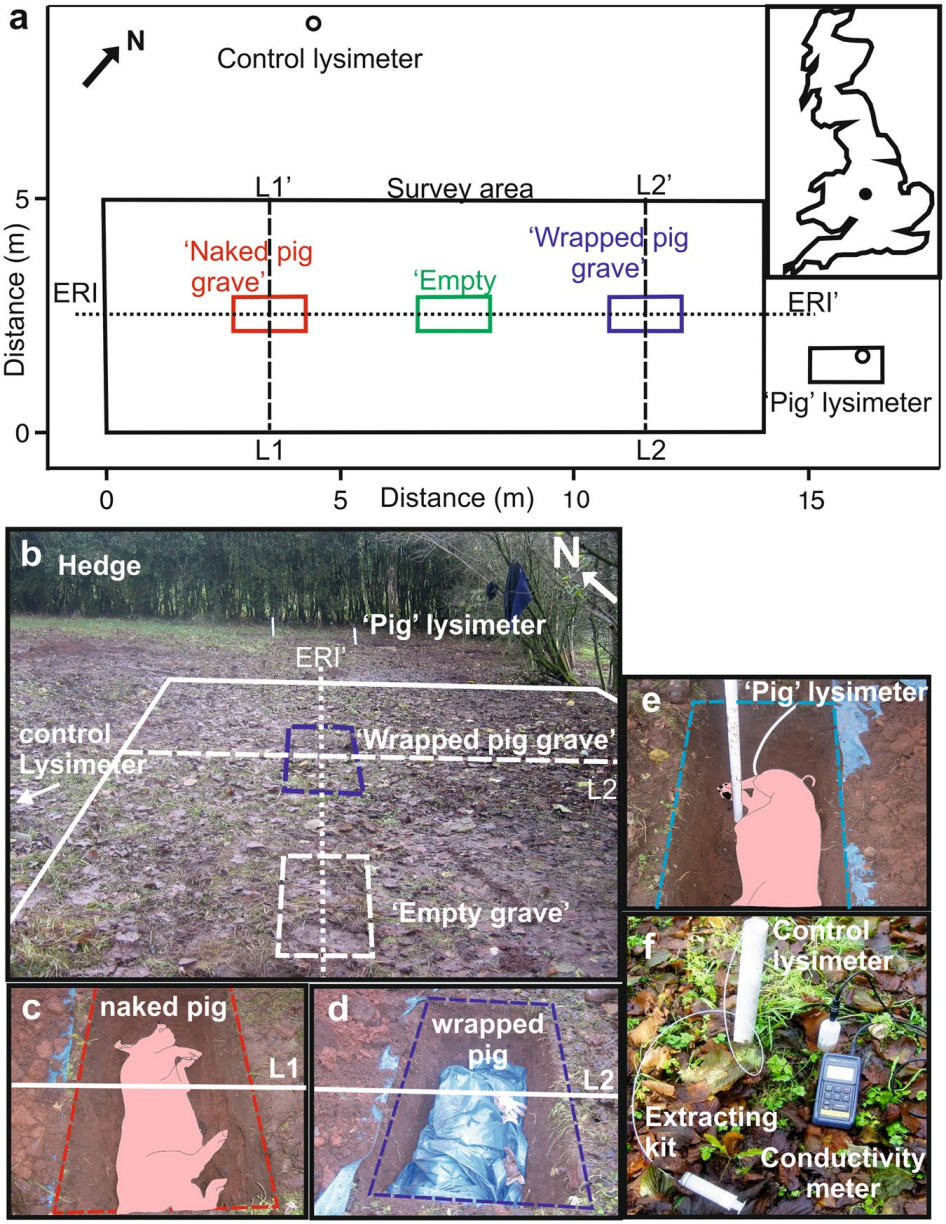
(**a**) Map of ERM survey area (rectangle) with graves, L1/2 GPR and ERI 2D profile lines, lysimeter soil water extraction positions and UK location map (inset). (**b**) Study site, (**c**) naked pig grave, (**d**) wrapped pig grave, (**e**) pig grave soil water and, (**f**) control soil water measurement photographs respectively. Modified from^14^.

The controlled test site was on Keele University campus, ~ 200 m above sea level, with a typical UK temperate climate^48^. The site was a grassed, rectangular area (~25 m × ~25 m), surrounded by deciduous trees (Fig. 2), with the geophysical survey area 5 m × 14 m and sloped by ~3° from northwest to southeast. The naked pig grave, an empty grave to act as control and the wrapped pig grave were in sandy loam soil (Fig. 2). Other relevant background site information is provided here^14^.

### Simulated graves

Four simulated graves were created in December 2007, with three used for geophysical surveys, one containing a naked pig carcass, one a pig carcass wrapped in woven PVC tarpaulin and the third empty grave for control (Fig. 2), and the fourth used for the electrical conductivity of grave soil water experiment. Pig cadavers are commonly used in decompositional research as they have similar chemical compositions, size, tissue:body fat ratios and skin/hair type to humans^15,50^. Both humans and pigs are omnivorous and therefore share a similar gut fauna^51^. The pigs weighed ~80 Kg each, and were sourced from a licensed abattoir, having been dispatched via a bolt gun on the frontal bone on the morning of grave deposition. The graves were ~1.5 m long, 0.75 m wide and 0.6 m deep and were separated from each other by ~5 m (Fig. 2). The grave emplacement procedure is described elsewhere^14^. Grave and control soil water composition was monitored via the installation of two lysimeters situated ~2 m and ~4 m from surveyed graves respectively (Fig. 2), with soil water conductivity regularly measured using a WTW multiline temperature-calibrated conductivity meter^48^.

### Electrical Resistivity Mapping (ERM)

Twin electrode (0.5 m fixed-offset) resistivity datasets were collected (Fig. 2) at three month intervals from years one to six and then annually in the winter to the end of the ten-year monitoring period (Supplementary Tables S1 and S2). Using the Geoscan Research RM15-D resistivity meter, readings were collected on a 0.25 m by 0.25 m grid, with remote probes placed at a fixed position 17 m away for consistency. Data were processed using Generic Mapping Tools v.5.4.3 software^52^. A minimum curvature gridding algorithm^53^ interpolated each dataset to 0.125 m × 0.125 m cell size. Long-wavelength trends were then removed to improve the identification of grave-sized anomalies by fitting a cubic surface to the gridded data and then subtracting this from the data. Seasonal changes in site conditions, particularly soil moisture content, cause variations in range of resistivity values ranges recorded at different times of the year^49^. Therefore, survey data were normalized by dividing each data set by its standard deviation. All resulting processed, normalized data sets had a zero mean value and standard deviation as units, allowing direct comparisons between the various resistivity survey data sets.

### Electrical Resistivity Imaging (ERI)

A 2D ERI survey line, orientated along the line of the three graves (Fig. 2), was surveyed at three month intervals for years 1–6 and annually to the end of the ten-year monitoring period (Supplementary Tables S1 and S3). 32 electrodes were placed at 0.5 m intervals along the 15.5 m long survey profile that bisected all three graves (Fig. 2a). Repeat ERI data acquisition used a Campus International TIGRE system and acquired using Campus Imager Pro v.2000 software. Raw ERI datasets were individually processed and inverted using a least-squares inversion approach using Geotomo Res2Dinv v.355 software following resistivity surveying recommendations^54^. The deepest four “n” levels were removed and half-cell spacing used to remove potential edge effects and reduce resistivity variations, respectively. DGPS survey data were also integrated to show topographic corrections. Final models of true resistivity sections were then created.

### Ground Penetrating Radar (GPR)

GPR 2D profiles were collected along two survey lines that bisected the two simulated pig graves (Fig. 2) at three month intervals for the first six years and then annually to the end of the survey period (Supplementary Table S1 and data S4). GPR data collection used Sensors&Software PulseEKKO 1000 equipment and 110 MHz, 225 MHz, 450 MHz and 900 MHz dominant frequency antennae, with radar trace separation being 0.2 m, 0.1 m, 0.05 m, and 0.025 m, respectively. Traces were stacked 32 times to increase the signal-to-noise ratio. Once GPR profiles were acquired, downloaded and imported into Sandmeier REFLEX-Win v.8.2.2 processing software, processing steps were applied to filter out noise and make reflection hyperbolae more pronounced. These steps were: (i) subtracting mean from traces “dewowing”, (ii) picking first arrivals, (iii) applying static corrections, (iv) applying 1D Butterworth bandpass filter and, (v) background removal to reduce any ringing effects.

### Meteorological information

The site was ~200 m from a weather observation station, which measured daily rainfall, air and ground temperatures plus soil temperature probes at 0.1 m, 0.3 m and 1.0 m below ground level. Monthly total rainfall and average temperature data over the ten-year study monitoring period were recorded (Supplementary Table S5). Total monthly rainfall during the period ranged from 2.6 mm to 166.6 mm, with an overall monthly average of 66.7 mm. Average monthly air temperatures ranged from −1.2 °C to 15.8 °C, with an overall monthly average of 6.5 °C (Supplementary Table S4). Accumulated Degree Day (ADD) data (see background^55^) weighted burial days by their respective daily average temperatures and summed them, which adjusted for site temperature fluctuations.

## Results

### Grave soil conductivity

**Figure 3:**
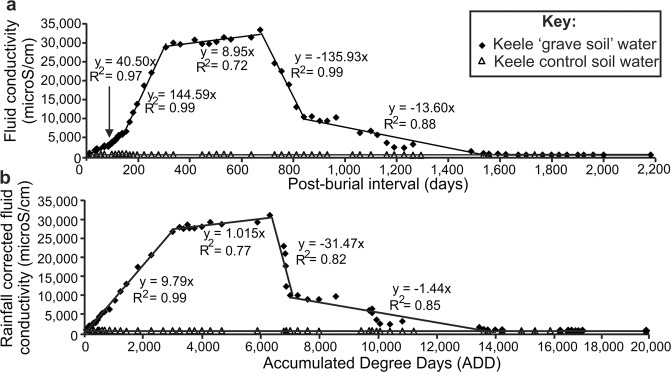
(**a**) Measured ‘grave soil’ water (diamonds) and control soil water (triangles) fluid conductivity values for the 6-year survey period. (**b**) Measured soil-water conductivity versus accumulated degree day (ADD) plot produced from (**a**) by summing average daily 0.3 m bgl temperatures (see^48^). Best-fit linear correlation formulae and good fit R^2^ values also shown. Meteorological and conductivity data is provided in Supplementary data Tables S1 and S2 respectively. Modified from^48^.

Control soil water measurements had consistent conductivity values (averaging 410 ± 0.1 mS/cm) over the six-year study period (Fig. 3). Grave soil water conductivity values rapidly increased from 265 ± 0.1 mS/cm (12 days) up to 28,800 ± 0.1 mS/cm (307 days) before gradually increasing to a maximum of 33,400 ± 0.1 mS/cm (671 days). Grave soil water conductivity values then rapidly decreased to 10,460 ± 0.1 mS/cm (840 days) before gradually decreasing to typical background values of 500 ± 0.1 mS/cm (1621 days) until the end of the six-year study period (2004 days). The grave soil water conductivity changes were grouped into six linear regressions with good fit R^2^ values of 0.72–0.99 (Fig. 3a). Site-specific temperature variations were also corrected for by converting post-burial days to Accumulated Degree Days (ADD) using the meteorological data (see Supplementary Tables S5 and S6), which improved R^2^ values for increasing conductivities (Fig. 3b), as it adjusts for the cadaver actively decomposing and producing fluid.

### Electrical Resistivity Mapping (ERM)

**Figure 4:**
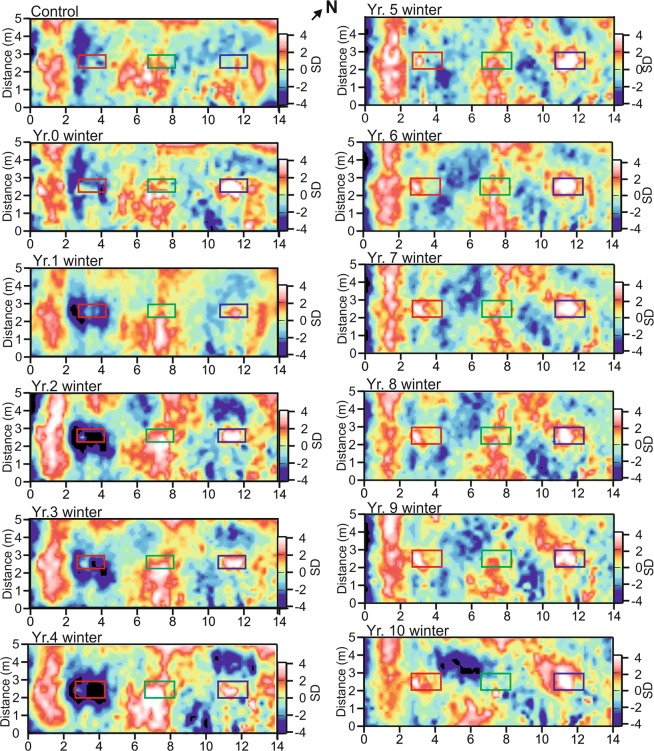
Electrode resistivity mapping (ERM) datasets (SD = Standard Deviation) for the ten-year (see labels) study period. Positions of respective naked pig (left), empty (center) and wrapped pig (right) graves shown (see Fig. 2a for site location). ERM data is provided in Supplementary data Table S3. Modified from^14^.

These surveys, mapping resistivity variations across the survey area containing the burials (see Fig. 2a) over the ten-year monitoring period, produced consistent results, with average resistivity values of 102.9 Ω (79.2 Ω minimum and 119.3 Ω maximum) and only ~2 anomalous ‘spike’ values per survey. Annual surveys are graphically contoured in Standard Deviations (SD) in Fig. 4 with data available in Supplementary Table S2. The empty control grave (central boxes in Fig. 4) could not be geophysically detected throughout the survey period. The naked pig grave (left boxes in Fig. 4) anomaly was temporally variable throughout the survey period. Up to year 4 it dominantly comprised a large negative anomaly (>−2 SD), then becoming a small amplitude positive anomaly (<0.5 SD) until the end of the survey period (Fig. 4). In contrast, the wrapped pig grave (right boxes in Fig. 4) showed predominantly a positive resistivity anomaly (<2 SD) up to year 4, broadly corresponding to the area of the grave, after which it increased in areal extent to the end of the survey period.

Analysis of the quarterly-acquired ERM surveys over the first six years of the survey period found a pronounced seasonal effect, with anomalies over the pig cadavers increasing in residual volume in winter and spring, and declining in summer and autumn (Supplementary Figure S1 available), with a continuous decline in size for the naked pig anomaly to the end of the survey period.

### Electrical Resistivity Imaging (ERI)

**Figure 5:**
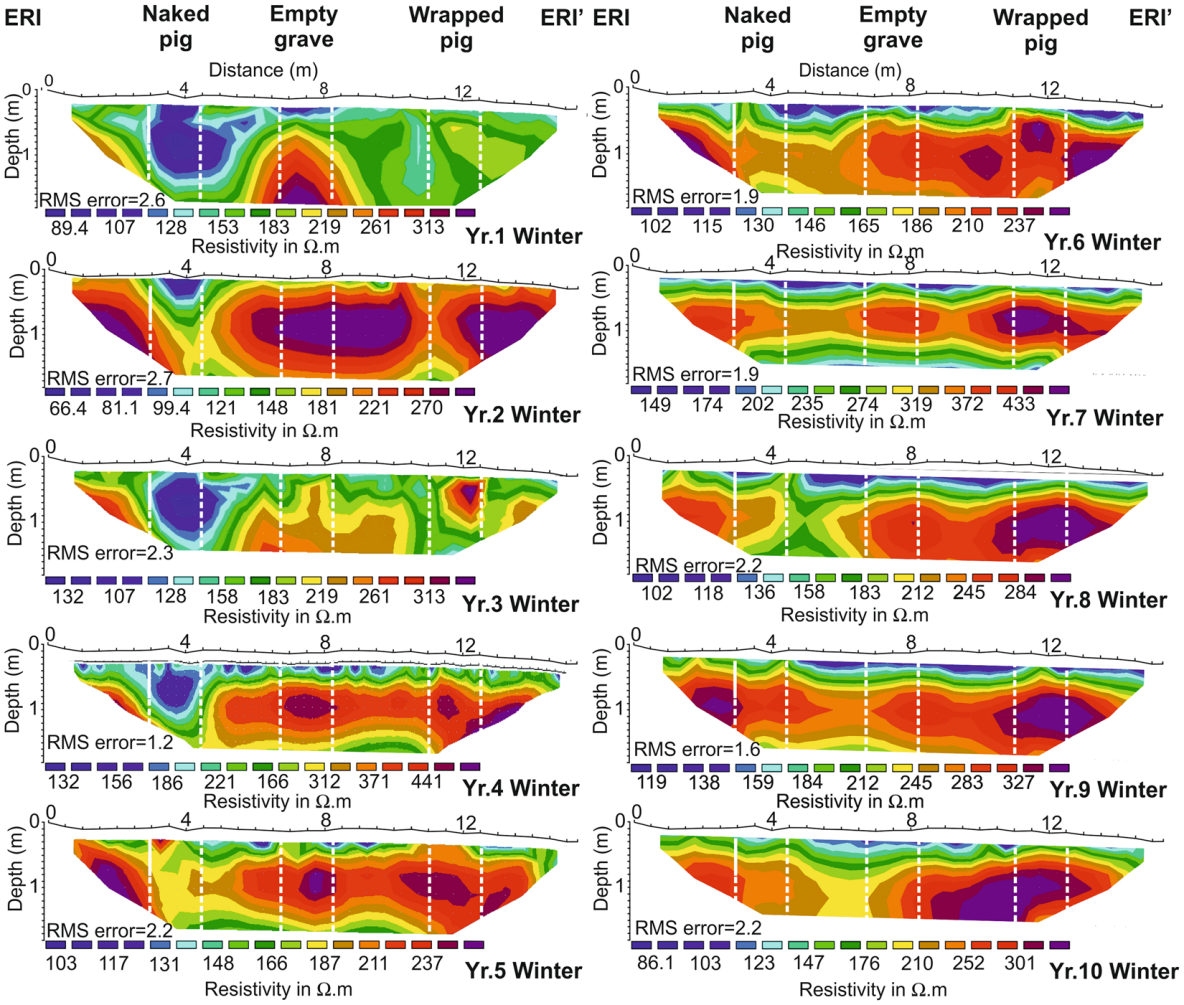
Electrical Resistivity Imaging (ERI) datasets for the ten-year study period (see labels), fifth iteration model inversion errors (RMS) shown (see text). Positions of respective naked pig (left), empty (center) and wrapped pig (right) graves shown (see Fig. 2a for site location). ERI data is provided in Supplementary data Table S4. Modified from^14^.

These surveys, mapping resistivity variations as a vertical slice through the area containing the burials (see Fig. 2a) over the ten-year monitoring period, produced consistent results, with average resistivity values of 203.5 Ω.m (157.3 Ω.m minimum and 269.9 Ω.m maximum). Inversion of the recorded data produced resistivity models with average RMS errors of around 2 after five iterations, which indicated very good fit between the data and models. Annual surveys are graphically shown in Fig. 5 with data available in Supplementary Table S3. The empty control grave (central boxes in Fig. 5) could only be detected as a negative resistivity anomaly in the first year of the survey period, after which it could not be detected to the end of the survey period. The naked pig grave (right boxes in Fig. 5), detectable as an anomaly, was temporally variable throughout the survey period. Up to four years from burial it was detectable as a consistent low resistivity anomaly, thereafter it was difficult to resolve to the end of the survey period (Fig. 5). The wrapped pig grave (left boxes in Fig. 5) was detectable as a small to large high resistivity anomaly, generally increasing in areal extent throughout the survey period (Fig. 5).

### Ground Penetrating Radar (GPR)

**Figure 6:**
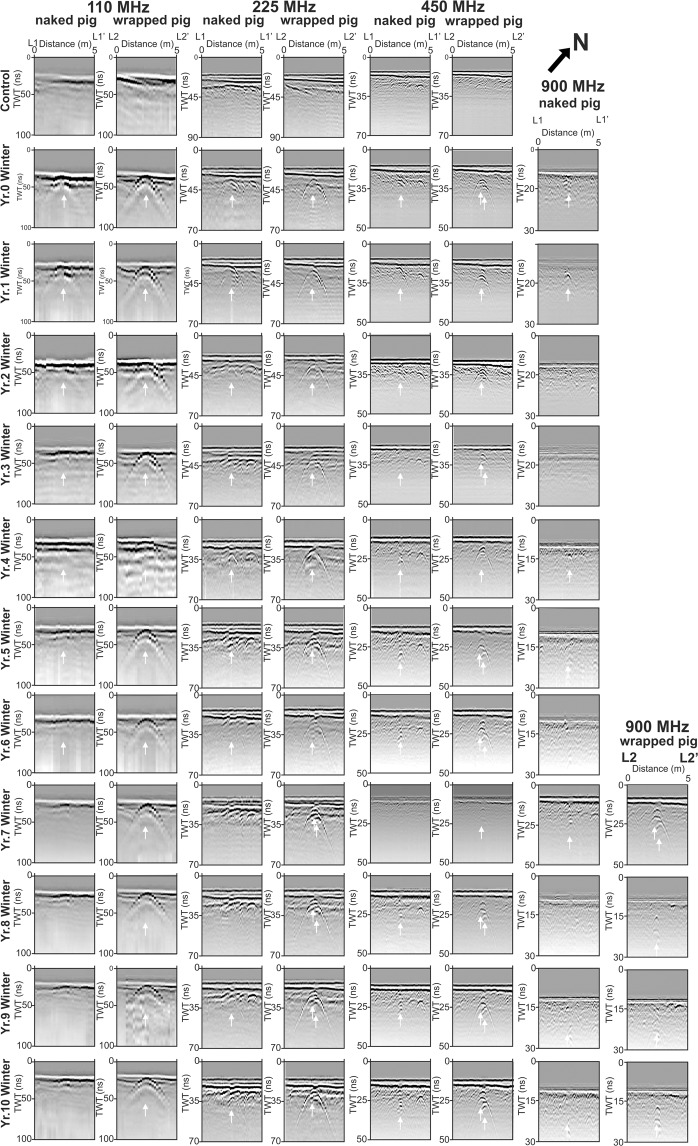
GPR 110, 225, 450 and 900 MHz frequency (left to right) 2D profiles for the ten-year monitoring period (survey dates on left) across the naked and wrapped pig graves (see labels) respectively (see Fig. 2a for site location). White arrows (where present) denote grave anomaly center location (see text). GPR data is provided in Supplementary data S5. Modified from^14^.

These surveys, imaging buried objects as a vertical slice through the area containing the burials (see Fig. 2a), were acquired over the ten-year monitoring period, with control data, collected before the burials were dug, showing no large buried objects were present prior to the start of the experiment (Fig. 6). Annual surveys are graphically shown in Fig. 6 with data available in Supplementary Data S4. The naked pig grave was poorly imaged as a low amplitude hyperbolic reflection at all frequencies up to year 6, and not detectable thereafter to the end of the survey period (Fig. 6). The wrapped pig grave was consistently and clearly detectable as a large hyperbolic reflection for all frequencies throughout the survey period, with a deeper reflection also observed in most GPR profiles (other than 110 MHz), probably generated from the base of the pig cadaver (arrows in Fig. 6). GPR anomaly amplitudes generally decreased throughout the survey period for all frequencies. There were also numerous small hyperbolic reflections events present in the medium to high frequency profiles not related to the graves, probably due to proximal tree roots or large stones. These would have made it difficult to identify a reflection from a grave if the position of these was not known *a priori*. Surveys parallel to the grave cut may produce larger anomalies; however, in the search for clandestine burials, the grave orientation is unknown so such surveys were not undertaken in this study.

## Discussion

This research is the first controlled forensic geophysical study involving the long-term monitoring simulated clandestine graves. Importantly, it uses both naked and wrapped cadavers, which represent the two main burial styles in discovered clandestine graves of murder victims^56^. This ten-year experiment allows forensic search teams to address fundamental questions that have not been resolved.

### Can electrical resistivity surveys and GPR successfully locate clandestine burials of homicide victims up to ten years after burial

Yes but this depends on the style of burial. Medium frequency (225/450 MHz) GPR surveys were shown in this study to be optimal, due to a combination of detectable anomalies over the survey period (other than from the naked burial), good target resolution and fewer false-positive features being imaged (Table 1). Additionally, such surveys are relatively rapid, when compared to higher frequency GPR surveys, which is important for forensic search teams if survey areas are large or personnel and/or budget are limited.

**Table 1:** Generalised summary of GPR and Electrical Resistivity (ERM and ERI) effectiveness to detect a homicide victim in a clandestine grave, based on this and other studies.

Geophysical method	Burial style	Post-burial period						
		Weeks	Weeks-Months	<1 Year	1–2 Years	2–3 Years	3–4 Years	4–10 Years
GPR low (110) frequency	Naked	●	●	●		○	○	○
	Wrapped	●	●	●	●	●	●	●
GPR medium (225–450) frequency	Naked	●	●					○
	Wrapped	●	●	●	●	●	●	●
GPR high (900) frequency	Naked				○	○	○	○
	Wrapped	●	●	●	●	●	●	●
Electrical resistivity mapping (ERM)	Naked	●		●	●	●	●	○
	Wrapped	●				●	●	●
Electrical resistivity imaging (ERI)	Naked	●	●			●	●	○
	Wrapped					●	●	●

Key: Good ●; Medium ; Poor ○; chances of success. Note this table does not differentiate between target size, burial depth / styles, other depositional environments (see^12,70^) and other important case-specific factors.

### How detectable was a buried naked cadaver over time?

The naked cadaver was not well detected in either low (110 MHz) or high (900 MHz) frequency surveys, and only poorly detectable at medium frequencies (225/450 MHz). This was comparable to similar, shorter timescale monitoring studies^16,41,42^, with the naked cadaver attenuating GPR signal as other researchers have noted^16,57^. This radar absorption would be exacerbated by the chest cavity collapsing during the later stages of decomposition (Fig. 1). Electrical resistivity surveys showed that a naked victim could be imaged for up to four years after burial (Figs. 4–5 and Table 1), due to the highly conductive body fluids producing a consistent negative resistivity anomaly (Fig. 3). This agrees with reports that buried remains require at least three years to reach advanced stages of decay, or skeletonization, at which point the majority of body fluids are lost and remains are considered dry^58^. However, this timing varies greatly and is dependent on the depositional environment and other variables such as burial depth, temperature, soil type and the presence of any clothing or wrappings^15,41,59^. Other research has shown that, if present, body-fluid conductivity could provide an indication of post-mortem interval (PMI) for a discovered clandestine grave^48^ which could be crucial for forensic investigators^60^. However, the current study shows that, after four years of burial, naked buried victims would be difficult to locate with electrical resistivity, as the majority of body fluids would migrate away from the victim’s cadaver and dissipate, which is especially problematic in sandy soils and rugged survey areas^1,30^.

### How detectable was a buried wrapped cadaver over time?

Wrapping a homicide victim prior to burial may help concealment in some ways (for example, it may trap scent reducing the effectiveness of victim recovery dogs and prevent decompositional fluids leaching into the soil that affects vegetation growth), but it does make a body easier to find using GPR. The wrapped cadaver was detectable on all GPR profiles (although 900 MHz antenna was not used until Year 7), due to the wrapping allowing stronger GPR reflections to be obtained. Research has shown that wrapping or clothing will slow decomposition rates^56^. A wrapped body left on the surface, perhaps whilst a grave is being prepared, will be protected from insect activity, the wrapping acting as a barrier to visiting blow flies and preventing them from ovipositing eggs on remains prior to burial^61^. This could result in smaller larval masses colonising and feeding on remains, reducing tissue loss and slowing rates of decomposition. Body fluids are also initially isolated from the surrounding soil and retained in or by the material^15^. Consequently, electrical resistivity imaging soon after burial should detect a small positive resistivity anomaly due to the higher conductivity of the wrapping compared to the surrounding soil. Victim body wrapping has been reported by others to slow decomposition^62^ and inhibit micro-organism activity^50^, which suggests this burial style may also be identifiable for a longer time post-burial, compared to a naked buried victim. Wrapping might also promote formation of adipocere, which would not only slow the rate of decomposition, but could preserve remains for years^63,64^. Adipocere, often referred to as “grave wax”, is a grey-white paste formed by hydrolysis and hydrogenation of adipose fats in a corpse^15^. Given the right conditions, a moist anaerobic environment with high bacterial activity, adipocere can appear within weeks following death but can persist for years. Over time the paste-like substance hardens to form a protective shell that preserves the remains, keeping them whole and intact^15^. Research has shown that buried wrapped pig cadavers experience significantly slower rates of decomposition due to the formation of adipocere^15,64^. Plastic sheeting, a tarpaulin, or even clothing, will accumulate fluid, reduce air flow and trap heat, creating an environment that promotes bacterial activity, and hence the formation of adipocere^15,64^. This could account for why the wrapped pig was more easily identified over a longer period, explaining the observation of deeper GPR hyperbolic reflection events apparent in the medium to high frequency profiles (multiple arrows in Fig. 6).

### When is the optimal time post-burial to do a forensic geophysical survey

From the results shown in this study and others^16,29,35^, irrespective of burial style (and vertical burials have been evidenced^65^), there is a general reduction in geophysical anomaly amplitude with increase in time since burial, so the sooner geophysical surveys can be undertaken the greater the chance of discovery (see Table 1 for general summary). Geophysical surveys should preferably be conducted prior to other, more invasive search methods, which may disturb the ground and introduce false-positive anomalies (e.g. metal detectors, soil/methane probes and victim recovery dogs). Geophysical surveys over much older human remains in graveyards/cemeteries have shown good results^37,56,58^, but the burial style is quite different (Fig. 1). The conductivity of grave soil water is highest one to two years post-burial (Fig. 3), which may be an important consideration for search teams when deciding on whether to include electrical resistivity surveys as part of their search strategy. Once positions of anomalies within the survey area are identified, these should be subjected to more detailed non-invasive scientific investigation, possibly including further geophysical surveys (e.g. 3D GPR grids), before intrusive investigations are undertaken, following a standard, phased investigative approach^3,4,12^.

### What effect does soil type have on forensic geophysical surveys

This study was on a sandy loam soil study site, with generally good results from GPR and electrical resistivity, although later surveys showed decompositional fluids dissipated into surrounding soil over time and therefore made locating naked burials harder. In other soil types, notably clay soils, fluids will be preferentially kept in grave soil, due to low permeability/porosity, and therefore electrical resistivity may be the optimal survey method, with generally poor GPR results reported from clay soils^12^. In contrast, good electrical resistivity results have been reported in other forensic studies in coastal sands^36^, chalky^26^, clay^32^ and black earth^66^ soil types, but relatively poor results reported in peat^1^ and coarse pebble soil types^66^.

### What effect does the seasonal timing of a survey have

Importantly there is also a seasonal effect in geophysical investigation, with winter and spring surveys generally having larger anomalies and better at resolving targets, when compared to summer and autumn surveys, shown here in Supplementary Figure S1 and by others^16,67,68^. Consequently, geophysical surveys in winter are suggested for forensic search teams, if operationally permitted, as these would have the best chance of victim detection success, as well as having less surface vegetation which may make surveying comparatively easier. For cold case searches, usually those which are either reviews or not active missing person searches, this would be the preferred option. These seasonal effects, especially in electrical resistivity, are due to soil having a reduced moisture content during the warmer and dryer periods inhibiting electrical current flow. In heterogeneous soil this effect is strongly non-uniform, introducing ‘noise’ within the geophysical data and masking the anomalies from any burials^67,68^. If there is a time-restricted element to the forensic search, then the optimal season of surveying should be ignored or an appropriate alternative search method^3,4,12^ chosen if necessary. Indeed, there may be particular combinations of burial and soil type and time since burial where geophysical anomalies may be enhanced in summer or autumn.

## Conclusions and Further Work

This study results and others^16,35,36,39^ should assist forensic search teams to use optimal search techniques and equipment configurations (see Table 1) and will be used to compare with active case data to improve detection rates.

A *clandestine burial of a naked homicide victim* should be detectable within the first four years of burial using electrical resistivity surveys in sandy soils, but then become progressively more difficult to locate. Other research^32^ shows resistivity surveys work in clay-rich soils due to highly conductive grave soil water being retained in grave soil. Medium frequency (225–450 MHz) GPR surveys are recommended. Winter and spring surveys have the highest chances of successful detection.

A *clandestine burial of a wrapped homicide victim* should be detectable for at least ten years after burial using electrical resistivity surveys. Medium frequency (225–450 MHz) GPR is again recommended due to a combination of good target resolution^16,44^; good penetration, few false-positive anomalies imaged and good data acquisition speed; however, low frequency (110 MHz) GPR also works, since the body wrapping produces a good reflective contrast. Again, winter and spring surveys are optimal.

The study will be continued to determine how long geophysical surveys will be able to locate these clandestine burials. Inorganic elemental analysis of grave soil water has determined potassium, sulphate and sodium as the major causes of electrical conductivity variations^69^, but it would be worth analysing what organic element changes are also occurring. Further analysis of geophysical results will also be undertaken to optimise surveys. This study should be repeated in other soil types and settings, for example, under cover, indoors and using more replicates so these can be dug up annually to evidence decomposition rates, and repeated using human cadavers to test the suitability of pig carcasses as analogues.

Although comparatively small in number, missing person cases are crucial for the victims’ families to solve to aid closure and to give confidence that justice will be served. This paper shows that buried victims are detectable for up to 10 years post burial, but winter/spring surveys have the best chance of successful detection.

## Supplementary information

Supplementary Information.

## Data Availability

Supplementary raw data accompanies this paper at: https://doi.org/10.21252/rvnp-1043.
