# Supplementary material for: Geophysical monitoring of simulated homicide burials for forensic investigations
Source: Sci Rep. 2020 May 5;10:7544. doi: 10.1038/s41598-020-64262-3 (PMC7200741; doi:10.1038/s41598-020-64262-3)
Supplement: Supplementary file 1 — Supplementary Information. [file 41598_2020_64262_MOESM1_ESM.docx]

**Geophysical monitoring of simulated homicide burials for forensic investigations**

Jamie K. Pringle^1*^, Ian G. Stimpson^1^, Kristopher D. Wisniewski^2^, Vivienne Heaton^3^, Ben Davenward^1^, Natalie Mirosch^3^, Francesca Spencer^4^, & Jon R. Jervis^1^


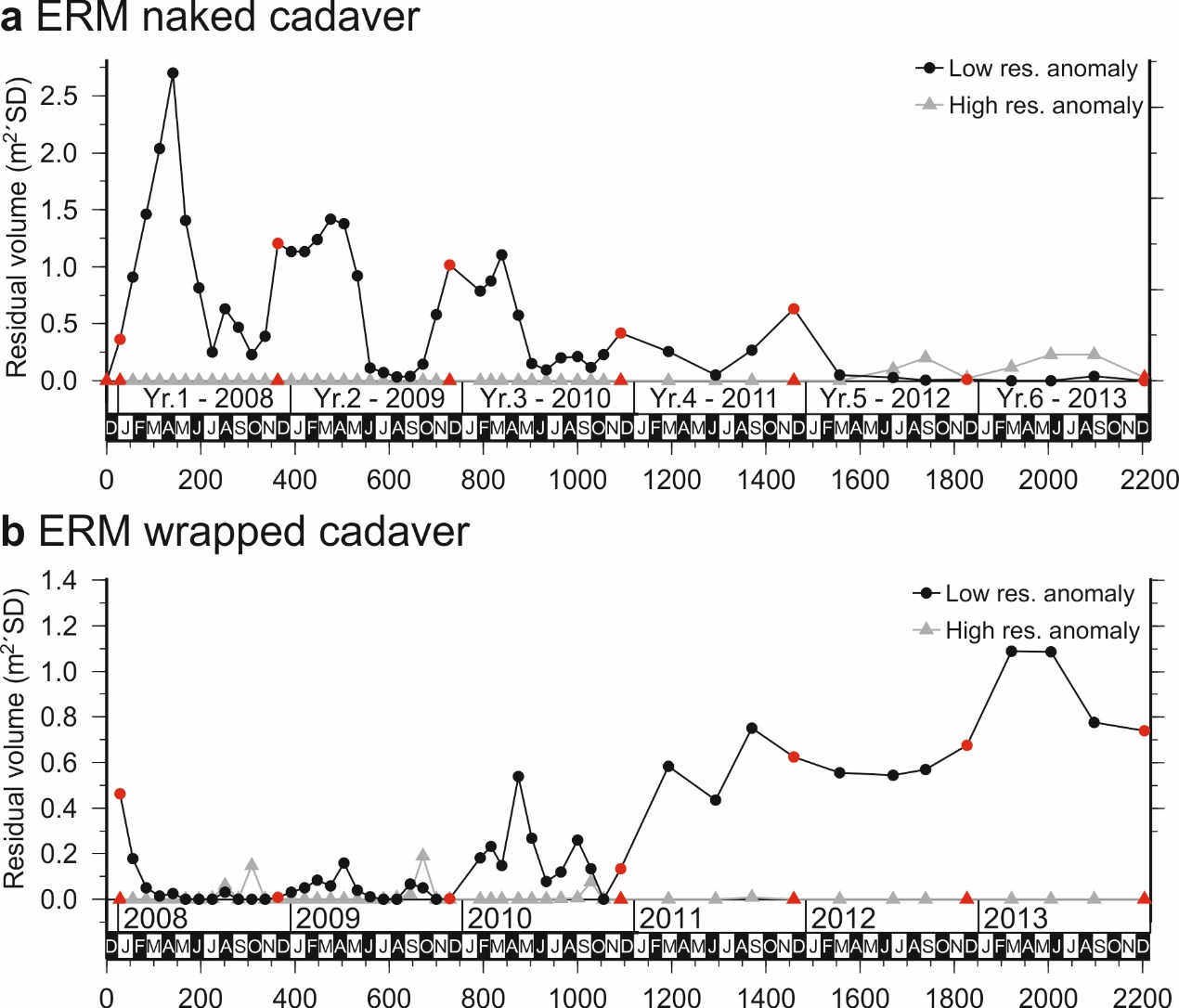


**Supplementary Figure S1.** Summary quantitative analysis plots of electrical resistivity mapping collected on the first six years of the survey period. Respective residual volume against years analysis for (**a**) naked pig and (**b**) wrapped pig cadaver. Red points indicate data shown in this paper. Modified from^48^.

Supplementary raw data accompanies this paper at: <https://doi.org/10.21252/rvnp-1043>

| **Survey details** | | | | **Resistivity datasets** | | | | **GPR datasets** | | | | | | | |
| --- | --- | --- | --- | --- | --- | --- | --- | --- | --- | --- | --- | --- | --- | --- | --- |
| **Year post-burial** | **Survey date** | **Survey day after burial^+^** | **Accum-ulated Degree Day*** | **ERM** | | **ERI** | | **110 MHz** | | **225 MHz** | | **450 MHz** | | **900 MHz** | |
|  |  |  |  | Naked pig | Wrap pig | Naked pig | Wrap pig | Naked pig | Wrap pig | Naked pig | Wrap pig | Naked pig | Wrap pig | Naked pig | Wrap pig |
| Year 0 | 19.12.2007 | 12 | 65 | 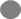 | 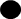 | N/A | N/A | 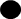 | 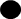 | 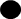 | 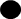 | 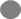 | 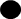 | 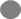 | N/A |
| Year 1 | 04.12.2008 | 363 | 3,732 | 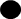 | 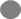 | 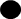 | 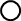 | 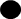 | 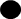 | 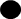 | 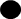 | 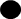 | 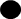 | 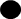 | N/A |
| Year 2 | 13.11.2009 | 707 | 7,371 | 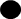 | 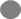 | 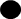 | 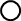 | 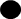 | 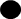 | 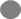 | 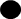 | 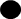 | 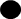 | 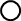 | N/A |
| Year 3 | 03.12.2010 | 1,092 | 11,026 | 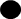 | 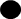 | 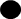 | 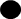 | 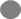 | 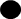 | 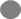 | 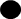 | 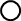 | 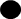 | 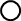 | N/A |
| Year 4 | 06.12.2011 | 1,460 | 14,827 | 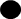 | 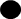 | 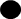 | 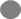 | 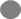 | 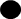 | 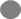 | 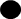 | 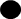 | 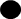 | 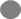 | N/A |
| Year 5 | 07.12.2012 | 1,827 | 18,636 | 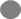 | 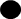 | 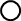 | 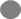 | 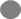 | 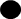 | 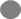 | 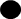 | 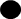 | 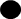 | 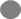 | N/A |
| Year 6 | 18.12.2013 | 2,204 | 22,345 | 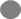 | 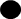 | 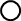 | 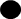 | 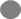 | 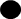 | 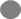 | 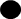 | 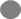 | 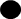 | 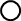 | N/A |
| Year 7 | 18.12.2014 | 2,568 | 26,503 | 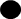 | 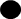 | 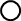 | 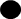 | 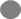 | 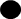 | 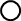 | 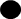 | 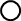 | 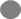 | 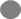 | 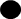 |
| Year 8 | 14.12.2015 | 2,929 | 30,336 | 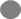 | 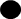 | 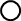 | 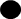 | 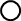 | 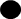 | 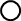 | 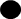 | 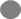 | 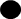 | 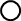 | 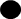 |
| Year 9 | 21.11.2016 | 3,272 | 34,273 |  |  |  |  |  |  |  |  |  |  |  |  |
| Year10 | 10.12.2017 | 3,656 | 38,477 |  |  |  |  |  |  |  |  |  |  |  |  |

**Supplementary Table S6.** Summary of annual geophysical surveys and respective anomalies (modified from^47^). ^+^Burial date was 7^th^ December 2007. *ADD based on average daily site temperatures at 0.3 m bgl (see^48^). Key: Good ; Medium ; Poor ; chances of success. N/A denotes data not collected. Table does not differentiate on target size, burial depth / styles, other depositional environments (see^12,71^) and other case-specific factors.
